# The definition of HIV-associated neurocognitive disorders: are we overestimating the real prevalence?

**DOI:** 10.1186/1471-2334-11-356

**Published:** 2011-12-28

**Authors:** Magnus Gisslén, Richard W Price, Staffan Nilsson

**Affiliations:** 1 Department of Infectious Diseases, Sahlgrenska University Hospital, University of Gothenburg, Sweden; 2 Department of Neurology, University of California San Francisco, San Francisco, CA, USA; 3 Department of Mathematical Statistics, Chalmers University of Technology, Gothenburg, Sweden

## Abstract

**Background:**

A substantial prevalence of mild neurocognitive disorders has been reported in HIV, also in patients treated with combination antiretroviral therapy (cART). This includes a new disorder that has been termed *asymptomatic neurocognitive impairment *(ANI).

**Discussion:**

ANI is identified by performance on formal neuropsychological testing that is at least 1 SD below the mean of normative scores in at least two cognitive domains out of at least five examined in patients without associated symptoms or evident functional impairment in daily living. While two tests are recommended to assess each domain, only one is required to fulfill this diagnostic criterion. Unfortunately, this definition necessitates that about 20% of the cognitively normal HIV-infected population is classified as suffering ANI. This liberal definition raises important ethical concerns and has as well diagnostic and therapeutic implications. Since neither its biological substrate, prognostic significance nor therapeutic implications are clearly established, we recommend that this diagnosis be modified or applied cautiously.

**Summary:**

The diagnoses of less severe forms of neurocognitive disorders in HIV relies on the outcomes of neuropsychological testing, and a high proportion of HIV-infected patients with effective cART may be classified as neurocognitively abnormal using the current criteria. The definition of ANI is not stringent, and results in approximately 20% of the population being classified as abnormal. To us this seems an unacceptable false-positive rate.

## Background

The introduction of combination antiretroviral therapy (cART) for human immunodeficiency virus (HIV) has had a substantial effect on morbidity and mortality in the HIV-infected population. Notably, the incidence of severe neurocognitive disorders, including AIDS dementia complex (ADC), also termed HIV associated dementia (HAD), has decreased markedly among HIV-infected patients during the last 15 years [1-3].

However, the prevalence of milder neurocognitive abnormalities detected by neuropsychological testing has been reported to remain high, including in patients treated with cART [4-7]. In 2007, a revised classification was proposed for HIV-related CNS impairment that included milder forms of neurocognitive disturbance [8]. This revised nomenclature, referred to as the *Frascati criteria*, established two new terms to cover milder impairment: *asymptomatic neurocognitive impairment *(ANI) and *minor neurocognitive disorder *(MND). Both are defined by impairment on neuropsychological tests compared to norms, but while the cognitive impairment is accompanied by mild interference in daily functioning in MND, ANI is by definition asymptomatic without such overt interference. ANI and MND are both subsumed within the broader term *HIV-associated neurocognitive disorders *(HAND), which additionally includes the more severe HAD [8] (Table 1).

**Table 1:** Criteria for HIV-associated neurocognitive disorders [8]

HAND	HIV-associated neurocognitive disorders
**ANI**	**HIV-associated asymptomatic neurocognitive impairment**
	Cognitive impairment involving at least two cognitive domains (performance of at least 1 SD below the mean for norms on neuropsychological tests)
	The cognitive impairment does not interfere with everyday functioning.
**MND**	**HIV-1-associated mild neurocognitive disorder**
	Cognitive impairment involving at least two cognitive domains (performance of at least 1 SD below the mean for norms on neuropsychological tests)
	The cognitive impairment produces at least mild interference in daily functioning
**HAD**	**HIV-1-associated dementia (HAD)**
	Marked cognitive impairment involving at least two cognitive domains (performance of at least 2 SD below the mean for norms on neuropsychological tests)
	The cognitive impairment produces marked interference with day-to-day functioning

High prevalence of ANI and MND has been reported in several recent studies using these new Frascati criteria. For example, Simioni et al found that 60% of HIV-infected subjects on suppressive cART and without any subjective complaints fulfilled the criteria of ANI [6], and 33% of patients without confounding comorbidities were diagnosed with ANI in the CHARTER-cohort [4]. While acknowledging that symptomatic mild neurocognitive impairment (MND) is a problem in some HIV-infected patients on cART, mainly in those with a history of low CD4 cell nadir, the very high prevalence of neurocognitive impairment in these reports does not correspond to our impression in everyday clinical experience. This prompted us to critically scrutinize the basis for ANI diagnosis.

## Statistical considerations

HAD is characterized by marked neurocognitive impairment ascertained by neuropsychological testing outcomes that are 2 standard deviations (SD) or greater below demographically-related means in at least two ability domains (attention-information processing, language, abstraction-executive, complex perceptual motor skills, memory, including learning and recall, simple motor skills and sensory perceptual abilities) [8]. Unlike HAD, ANI and MND are defined by performance at least 1 SD below the mean of normative scores in at least two cognitive areas; these criteria specify that at least five of the cognitive domains are examined or observed. If possible, at least two tests per domain are recommended; however, this is not mandatory, in many settings is impractical and has not been followed rigidly in all reports [9]. If two or more tests are used per domain not all individual tests are needed to fall below 1 SD of appropriate norms. Impairment in only one of the tests has been sufficient in some studies [6] while the mean results or some other aggregating method has been used in others [4]. The latter leads to similar statistical considerations as with one test performed for each domain. As mentioned before, MND differs from ANI in that the cognitive impairment produces at least mild interference in daily functioning. The HAND diagnoses are applied only if the cognitive impairment cannot be explained by other comorbidities, though this is sometimes difficult and one report divides subjects into those with incidental, contributing and confounding comorbidities - distinctions that may be arbitrary and difficult to apply in many patients [4].

Providing normal distribution, 2.3% of a population will perform worse than 2 SD below the mean and 15.9% below 1 SD on a given test. Thus, in each neuropsychological test almost 16% of the population will have a test result that is defined as abnormal. The probability of a test result below 1 SD in at least two tests depends on how many tests are performed and also how these tests intercorrelate. Despite assessing different domains, a positive correlation between some tests of neuropsychological performance can be presumed, though the magnitude of this correlation is uncertain, i.e. the probability to perform abnormally when testing one domain is increased if the test performance of another domain is abnormal.

**Figure 1:**
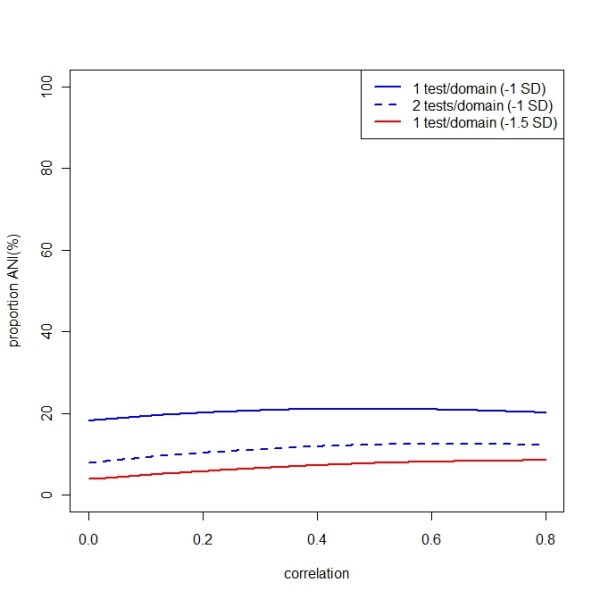
**Probability of a normal control to be categorized as abnormal according to the criteria of asymptomatic neurocognitive impairment (ANI) and minor neurocognitive disorder (MND) if five cognitive domains are tested**. Defined as impairment in functioning, involving at least two ability domains, documented by performance of at least 1.0 SD below the mean for norms in one test per domain (blue solid line), in two tests per domain (blue dotted line) or of at least 1.5 SD below mean for norms in one test per domain (red line). The probability is plotted as a function of the degree of correlation between test results of different cognitive domains with using 0.8 as correlation between tests in the same domain.

Assume the tests of five domains follow a multivariate normal distribution and that the correlations between tests in the same domain are 0.8, while the correlations between tests in different domains are 0 ≤ ρ ≤ 0.8, i.e. the same non-negative correlation ρ. While the theoretic calculation of the probability for ANI is non-trivial for arbitrary ρ, it can easily be determined by simulations. Figure 1 shows a probability plot with various correlations. The probability varies between 18 and 21% with performance worse than 1 SD below mean in one test (or average of tests) per domain on at least two domains of five tested (blue solid line). If more tests or domains are assessed [4,6], the probability of an abnormal test result will be even higher. The reality is however more complicated, and there are likely differing correlations between each of the tests; however, the probability will never be lower than 16%. In fact, this corresponds well to what has been noted in normals using these cutoffs [4]. If more than 1 SD impairment in two tests per domain are required, the probability to be diagnosed as impaired will be lower, between 8 and 13% and if 1.5 SD are used as cut-off (Figure 1, blue dotted line), the probability will be 4-8% to perform abnormal, with one test per domain (Figure 1, red line).

## Discussion

The diagnoses of less severe forms of HAND relies on the outcomes of neuropsychological testing, and a high proportion of HIV-infected patients with effective cART may be classified as neurocognitively abnormal using the Frascati criteria, most commonly with mild deficits, i.e. ANI and MND. In addition to the problem that the comparison group in evaluating neuropsychological testing results consists of *normals *who were presumably screened for confounding conditions rather than background-matched *controls *tested in the same setting, the definition of ANI is not stringent, and results in between 16 and 21% of the population being classified as abnormal. To us this seems an unacceptable false-positive rate.

There are several problems with using such a generous definition. First, by exaggerating the prevalence by a loose definition, the real extent of inapparent HIV-related brain disease is obscured. There is little doubt that some patients, often long-term infected and treated, with complaints about milder memory problems and slowness, difficulties in concentration, planning, and multitasking suffer impaired cognitive function. Some of these have no confounders or alternative explanations and presumably represent development of brain injury either before or during suppressive cART. In the latter case, this may relate to ongoing HIV replication within the CNS despite controlled infection in the periphery [10]. More common may be mild neurocognitive impairments as a consequence of HIV-related CNS injury in the past, before treatment initiation. This is suggested by the fact that low blood CD4 cell nadir is a risk factor for neurocognitive impairment during cART [11]. However, augmenting the prevalence of these disorders by false-positive identification of impairment confounds both clinical trials and daily clinical management.

Second and importantly, there is an ethical dilemma in categorizing patients that don't have any symptoms or complaints as neurocognitively impaired. There is no evidence that patients with ANI are at increased risk to develop more severe impairment or have a need of any specific intervention. This is especially true in patients with an ongoing effective antiretroviral treatment. An ANI diagnosis, with uncertain relevance, may also lead to anxiety and impact on that person's life and employment.

Similar considerations of early detection of neurocognitive impairment are also discussed in other diseases. In contrast to ANI, the definition of mild cognitive impairment (MCI) as a precursor for Alzheimer's disease requires subjective memory complaints in addition to impaired neurocognitive performance [12]. In Alzheimer's disease, advances in the understanding of imaging and biochemical changes occurring early in the illness have improved the ability to diagnose the disease in its early phase. Biomarkers are now incorporated in the diagnostic criteria of Alzheimer's disease [13] and may even be useful in predicting incipient development of Alzheimer's disease in those with MCI [14].

The uncertain incidence of ongoing brain injury during antiretroviral treatment, indicates a need for a more biologically-based and precise definition of abnormality also in HIV-associated neurocognitive disorders. Such a definition should preferably include objective laboratory- based biomarkers in addition to neuropsychological testing [15]. We also suggest that the definition of abnormality in neurocognitive performance of asymptomatic HIV-infected patients without neurocognitive complaints should be modified, for example by changing the cut-off of individual tests or preferably the mean domain performance to below 1.5 SD to lower the false-positive rate.

This is not simply a theoretical issue, but one that has important therapeutic implications. If one is to target continued persistent neurological impairment, it is critical to first define whether the impairment is indeed genuine and not just part of a more than 16% false-positive rate consequent to the nature of the definition involving 1 SD as cut off in neuropsychological testing, and then to separate active indolent injury from past injury.

## Summary

The diagnoses of less severe forms of HAND relies on the outcomes of neuropsychological testing, and a high proportion of HIV-infected patients with effective cART may be classified as neurocognitively abnormal using the current criteria. The definition of ANI is not stringent, and results in between 16 and 21% of the population being classified as abnormal. To us this seems an unacceptable false-positive rate.

## Conflicts of interests

The authors declare that they have no competing interests.

## Authors' contributions

All authors conceived of the idea for the report. SN prepared the statistics. All authors contributed to revision and approved the final report for submission.

## Pre-publication history

The pre-publication history for this paper can be accessed here:

http://www.biomedcentral.com/1471-2334/11/356/prepub
